# Prognostic significance of c-KIT in vulvar cancer: bringing this molecular marker from bench to bedside

**DOI:** 10.1186/1479-5876-10-150

**Published:** 2012-07-28

**Authors:** Beatriz de Melo Maia, André Mourão Lavorato-Rocha, Iara Sant’Ana Rodrigues, Glauco Baiocchi, Flávia Munhoz Cestari, Monica Maria Stiepcich, Ludmila Thomé Domingues Chinen, Kátia C Carvalho, Fernando Augusto Soares, Rafael Malagoli Rocha

**Affiliations:** 1 Anatomic Pathology Department, Hospital AC Camargo, Rua Antônio Prudente, 109. 1o Andar–Patologia Investigativa, Liberdade, São Paulo, SP, CEP: 01509-900, Brazil; 2 Gynecologic Oncology Department, Hospital AC Camargo, São Paulo, Brazil; 3 Pathology Department, Fleury Institute, São Paulo, Brazil; 4 Oncology Department, Hospital AC Camargo, São Paulo, Brazil; 5 Obstetrics and Gynecology Department, School of Medicine of Sao Paulo University, São Paulo, Brazil

**Keywords:** Vulvar carcinoma, HPV, c-KIT, Immunohistochemistry, qRT-PCR

## Abstract

**Background:**

Vulvar carcinomas are rare tumors, and there is limited data regarding molecular alterations. To our knowledge there are no published studies on c-KIT and squamous cell carcinomas of the vulva (VSCC). Although there are a significant number of other tumor types which express c-KIT, there remains controversy as to its relationship to patient outcome. Thus, we wished to investigate such controversial findings to determine the prognostic importance of c-KIT by evaluating its protein and mRNA expression in VSCCs, correlating these findings with clinicopathological features and Human Papillomavirus (HPV) infection.

**Methods:**

c-KIT expression was scored by immunohistochemistry (IHC) as positive or negative in 139 formalin-fixed paraffin-embedded (FFPE) cases of vulvar carcinomas arrayed in a tissue microarray (TMA) using the DAKO® A4502 rabbit polyclonal c-KIT antibody (diluted 1:100). *c-KIT* mRNA was evaluated by qRT-PCR in 34 frozen samples from AC Camargo Hospital Biobank (17 tumoral and 17 non-tumoral samples) using TaqMan probes–Applied Biosystems [Hs00174029_m1]. HPV genotyping was assessed in 103 samples using Linear Array® HPV Genotyping Test kit (Roche Molecular Diagnostics, Basel, Switzerland). All results obtained were correlated with clinical and pathological data of the patients.

**Results:**

c-KIT protein was positive by immunohistochemistry in 70.5% of the cases and this was associated with a higher global survival (p = 0.007), a higher recurrence-free survival (p < 0.0001), an absence of associated lesions (p = 0.001), lymph node metastasis (p = 0.0053), and HPV infection (p = 0.034). Furthermore, *c-KIT* mRNA quantitation revealed higher levels of transcripts in normal samples compared to tumor samples (p = 0,0009).

**Conclusions:**

Our findings indicate that those vulvar tumors staining positively for c-KIT present better prognosis. Thus, positivity of c-KIT as evaluated by IHC may be a good predictor for use of more conservative surgery techniques and lymph node dissection in vulvar cancer. So part of the essence of our study is to see the possibility of translating our current results from the bench to the bedside. This will help provide patients a more appropriate, less mutilating treatment, in order to keep the maximum physical and psychic quality as possible to these women.

## Background

Vulvar carcinoma is a rare malignant tumor that presents most commonly as a squamous cell carcinoma (90% of all cases). This neoplasm comprises 3–5% [1-3] of female genital tract malignant tumors, traditionally being considered a disease of elderly women (aged between 65 and 75 years) [4,5]. However, in recent decades, its incidence has risen in younger women, [6] possibly due to the higher frequency of precursor lesions–vulvar intraepithelial lesions (VINs)–and their association with Human Papillomavirus (HPV) infection [6,7]. The medical interest in this type of carcinoma was accentuated in the last decade due to the recognition of the increasing incidence of the disease. Nevertheless, in spite of the increasing interest, limited knowledge of molecular alterations in this type of tumor means that translational research is almost absent in this field.

The *c-KIT* proto-oncogene, also known as SCFR or CD117 is the v-kit Hardy-Zuckerman 4 feline sarcoma viral oncogene homolog. Situated at the long arm of chromosome 4 at 4q11.12, this gene is physiologically expressed in melanocytes, germ cells [8,9] tissue mast cells, mammary epithelial cells and sudoriferous glands and skin basal layers cells [9-11]. During development and adulthood, *c-KIT* modulates various processes including survival, proliferation, migration and differentiation of hematopoietic cells, germ cells and cells derived from the neural crest [12,13].

**Figure 1:**
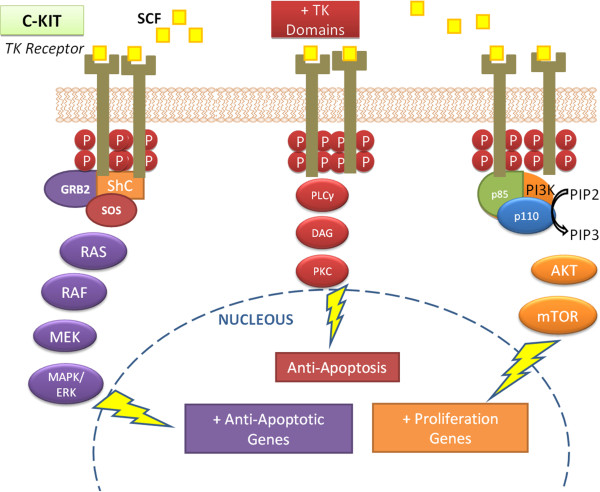
**Main pathways activated by the tyrosine-kinase receptor c-KIT and its effects on carcinogenesis.** KIT dimerization occurs from the SCF (yellow) coupling, which leads to phosphorylation of the tyrosine kinase domains (shown with red dots along the intracellular portions of receptors), which triggers the coupling and recruitment of several intracellular proteins that in turn, activate the three routes shown in colors (MAPK, PLCγ and PI3K pathways in purple, red and orange, respectively). This process culminates in cellular activities highlighted in rectangles.

A member of the Tyrosine Kinase Receptor Family III (β-PDGFR/CSF-1 Family), *c-KIT* encodes a transmembrane receptor and has a specific ligand called SCF (stem cell factor, also known as c-KIT ligand or mast cell growth factor) [9]. SCF has two alternatively spliced forms: whereas the soluble form immediately stimulates tyrosine kinase activity, the membrane-bound form produces long-term effects, increasing the stability of the receptor and preventing its rapid downregulation [9]. The interaction between SCF and c-KIT receptor activates the tyrosine kinase function which, in turn, induces phosphorylation of various cellular proteins and activation of a number of cascades, among them via RAS, PI3K and PLCγ, as shown on Figure1. 

Various types of neoplasms express c-KIT. In GISTs, for example, this expression is very common [14]. Also, angioleiomyolipomas [15], small-cell lung carcinomas, seminomas/dysgerminomas [10], and leukemias [9,16] frequently express c-KIT. In many tumor types, this receptor is associated with malignancy. In gastrointestinal stromal tumors, myeloid leukemias and mast cell disorders, for example, c-KIT gene gain-of-function mutations result in constitutive tyrosine kinase activity and are considered to play a central role in oncogenesis and sustained tumor growth [17,18]. Coexpression of c-KIT and its ligand in small-cell lung cancer, for example, appears to result in an autocrine growth loop sustaining tumor cell proliferation [9,19]. On the other hand, a markedly better outcome has already been demonstrated in tumors that expressed c-KIT compared with those that did not, such as neuroblastomas [9], nasopharyngeal carcinomas [20] and multiple myeloma [21].

Such controversial findings lead us to investigate the role of this receptor in vulvar carcinomas, as the literature is very limited in this respect and, to our knowledge, no study has been published regarding c-KIT evaluation in squamous cell carcinomas of the vulva (VSCC). For this reason, the present study was designed to evaluate whether c-KIT predicts patient’s outcome in VSCCs and, if so, if this marker can be considered as an efficient prognostic factor. We assessed mRNA expression of c-KIT and its protein product in vulvar squamous cell carcinomas and correlated these data with clinicopathological features and with the presence of HPV infection, in order to determine the prognostic importance of this receptor in vulvar cancer.

## Methods

### Patients and samples

This was a retrospective study of 139 formalin-fixed paraffin-embedded (FFPE) tumor tissue samples selected retrospectively and randomly of patients who had surgical intervention–biopsy or vulvectomy–selected from the Anatomic Pathology Department of A.C. Camargo Hospital, São Paulo, Brazil between the years 1979 to 2006.

According to standardized protocols (used in this institution) all tumor specimens obtained from surgery or biopsy are always maintained in 10% buffered formalin for 8 hours and then paraffin-embedded and enclosed in a labeled cassette for storage in the archives of the Anatomic Pathology Department. For our study, diagnosis was confirmed and cases were re-classified by an experienced pathologist. All cases were arrayed in duplicates by selecting representative areas of tumor arranged in a tissue microarray (TMA), using the tissue arrayer (Beecher Instruments, Silver Springs, Maryland). In situ carcinomas, patients who received neoadjuvant treatment (chemotherapy or radiotherapy) and those tumors whose paraffin blocks were unavailable or contained insufficient material were excluded from the study. Furthermore, 17 frozen tissue samples of surgically removed tumors and 17 non-tumor samples adjacent to the tumor stored in the A.C. Camargo Hospital Biobank, were used to assess mRNA expression by qRT-PCR. This work has been approved by the Ethics Committee of our institution (AC Camargo Research Ethics Committee–Number 1379/10) and is in compliance with the Helsinki Declaration.

### Immunohistochemistry

IHC was performed in TMA section using the automated Ventana Benchmark® platform (Ventana Medical Systems, Inc., Tucson, Arizona). DAKO® A4502 rabbit polyclonal c-KIT antibody diluted 1:100 was used; positive and negative controls were included in the batch.

### Assessment of immunoreactivity

The immunoreactivity of c-KIT protein was evaluated on the cell membrane and/or cytoplasm, graded as previously described by other authors [22], based on two parameters: intensity of staining and percentage of stained cells. The intensity of staining was scored as 0 = negative, 1 = weak staining, 2 = moderate staining, 3 = strong staining. The percentage of labeled cells was scored as (a) sporadically stained = less than 10% of positive cells, (b) focal staining = amount greater than or equal to 11% and less than 50% of the stained cells, and (c) diffuse = more than 50% of cells positively stained. Positivity cut off was established with staining intensity of 2 + or 3 + combined with focal or diffuse pattern, respectively.

### Quantitative RT-PCR

Total RNA was extracted from 17 frozen tissue samples of surgically removed tumors and 17 non-tumor samples (adjacent to the tumor) using the RNeasy Mini Kit (QIAGEN, Austin, TX, USA), according to the manufacturer’s protocol. cDNA was synthesized from total RNA using the High-Capacity cDNA Reverse Transcription Kit (Applied Biosystems, Foster City, CA, USA). Quantitative RT-PCR was performed in Applied Biosystems 7900HT Fast Real-Time PCR System (Applied Biosystems, Foster City, CA, USA). A set of primers and TaqMan probes for *c-KIT* were purchased from Applied Biosystems [Hs00174029_m1]. A pool of normal skin tissue was used as the control sample and *HPRT* was elected as the reference gene. The relative quantification (RQ) method was applied to determine the gene expression levels. Values of RQ within the range (RQ ± 2SD) in the corresponding referent group were accepted as normal.

### HPV DNA detection and genotyping

DNA was obtained and isolated from 139 formalin FFPE tissues following the supplier’s recommendations for QIAamp DNA FFPE Tissue Kit, based on the QIAamp method (QIAGEN, Valencia, CA, USA). One hundred and three samples demonstrated satisfactory quality of the DNA obtained and were used for the HPV genotyping using the Linear Array® HPV Genotyping Test kit (Roche Molecular Diagnostics, Basel, Switzerland). The results of the hybridization were assessed visually by comparing with the standard grid. Absorbance readings greater than 0.2 were classified as positive for either HPV and/or β-globin presence.

### Statistical analysis

The significance of the associations between IHC results with histological features and clinical data was analyzed by Fisher’s exact test adopting p < 0.05 as the significance level. Kaplan-Meier analysis was performed for survival curves using the log-rank-test and the date of diagnosis to the death or last follow up. *Software SPSS* for Windows (version 13.0) was used for statistical analyzes.

## Results

### Demographic, anatomopathological and clinical data of the patients

The mean age of the onset of the disease was 69 years (SD = 12), ranging from 15–98 years and median age of onset was 71 years. Most patients ranged in age between 70 and 80 years (54.67%). Regarding demographics, the majority of patients were white (86.3%), married (50.30%), and almost all women (78.37%) were illiterate or had incomplete primary education.

Most tumors were classified as well-differentiated Squamous Cell Carcinomas (SCC)–SCC1 (43.88%), followed by SCC 2 (33.81%), SCC 3 (10.07%), basaloid carcinomas (9.35%) and verrucous and sarcomatoid carcinomas (1.44% each). In relation to the depth of invasion, 20.78% infiltrated the superficial dermis, 54.55% of the tumors infiltrated the deep dermis and 24.68% infiltrated the subcutaneous tissue. A moderate inflammatory infiltrate was most commonly observed (62.6%). Most tumors showed no vascular invasion (84.89%) or perineural invasion (89.21%). No lymph nodes metastases were observed in 71.94% of cases which had had a lymphadenectomy. However, 28.06% had some degree of lymph node involvement. Among our series, most of tumors were classified as FIGO staging IB (58.91%) and IIIB (19.37%), according to the staging proposed by the International Federation of Gynecology and Obstetrics (FIGO).

Treatment complications were found in 54.5% of women, and recurrence was observed in 38.58% of cases: local and nodal recurrences were more frequent (38.78% and 24.49% of all types of recurrence, respectively). Among women with any recurrence, 65.3% died of this cancer. In relation to the overall status of the patients, 41.30% were alive without cancer, 33.33% died of the disease, 13.04% died of other causes, 7.24% were alive with disease, and 5.07% died of unspecified causes. Global survival for all patients showed a median of 86 months.

### c-KIT immunostaining

**Figure 2:**
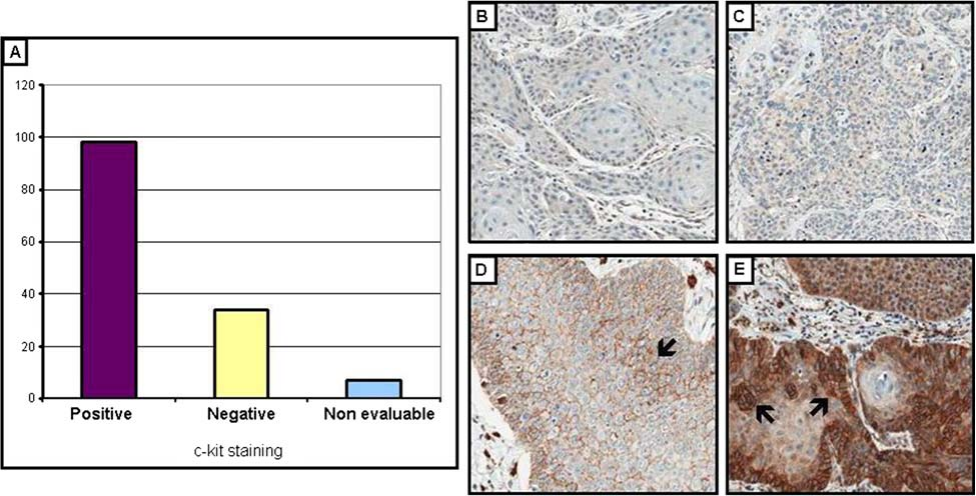
**c-KIT immunostaining in vulvar carcinomas: evaluation of immunoreactivity at the membrane and/or cytoplasm (200x magnification).** (**A**) Graphical plot of c-kit immunostaining results by IHC: the number of samples is shown in vertical axis, and the staining in the horizontal axis; (**B**) and (**C**): Negative immunostaining for c-KIT; (**D**) Positive immunostaining for c-KIT in the membrane (arrow); (**E**) Positive immunostaining for c-KIT in the membrane and cytoplasm (arrows).

Positivity of c-KIT by immunohistochemistry was observed in the cell membrane and/or in the cytoplasm of 70.5% of the cases (98/139 cases). Almost 24% of the cases were negative and 5.04% (7 cases) were not evaluated as the TMA spot was missing. Positivity and negativity of c-KIT are illustrated in Figure2.

### Protein expression and clinicopathological features

Associations between c-KIT expression and clinicopathological features and HPV infection are listed in Table1. 68% of c-KIT positive tumors did not show any type of associated lesions, such as in situ carcinoma, VINs, lichen sclerosus or vulvar acanthosis (p = 0.0001). c-KIT positive cases were associated with an absence of, or only one, lymph node involvement (p = 0.0053) when compared to c-KIT negative cases, which were associated with two or more lymph-node metastasis.

**Table 1:** Evaluation of histopathological characteristics, clinical data and HPV infection of the patients in relation to c-KIT immunostaining

**c-KIT immunostaining**					
**Variables**	**Category**	**Staining**		**Total**	**P value**
		**Negative**^**a**^	**Positive**^**b**^		
		**n (%)**	**n (%)**		
***HPV***	Negative	17 (30)	6 (15)	24	0.034
	Positive	40 (70)	34 (85)	75	
***Nodal metastasis***	*No*	9 (47)	35 (69)	44	0.0053
	*Yes*	10 (53)	16 (31)	26	
***Associated lesions***	*No*	26 (84)	62 (68)	88	0.0001
	*Yes*	5 (16)	29 (32)	34	

**a.** Negative: no immunostaining.

**b.** Positive: membrane and/or cytoplasm staining.

### c-KIT expression and HPV

HPV infection was detected in 58.25% of the samples. Among those, HPV16 (44.18%) and HPV33 (27.9%) were the most commonly found. 77% of the patients presented only one infecting type of the virus. However, mutual infection by two or three types of HPV was observed in 11.62% of cases, each (Table1). There was a significant agreement between c-KIT positivity and HPV infection (p = 0.034).

### c-KIT expression and patient’s survival

**Figure 3:**
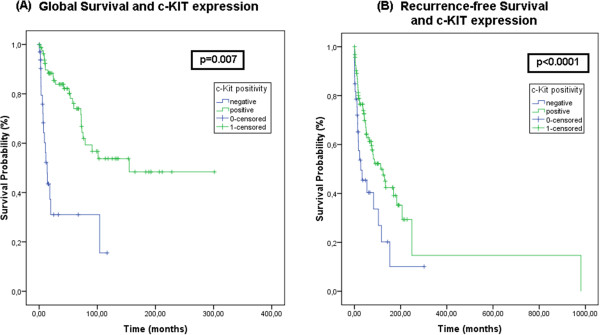
**Kaplan-Meier Global and Recurrence-free survival curves for c-KIT staining.** c-KIT positivity correlated with better global survival (p = 0.007) (**A**) and recurrence-free survival (p < 0.0001) (**B**) when compared to negative stained tumors (shown in blue lines).

Our results showed a better 5 year global survival (p = 0.007) and recurrence-free survival (p < 0.0001) for patients with c-KIT positive tumors compared to those whose tumors were negative, as shown in Figure3.

### c-KIT mRNA expression

**Figure 4:**
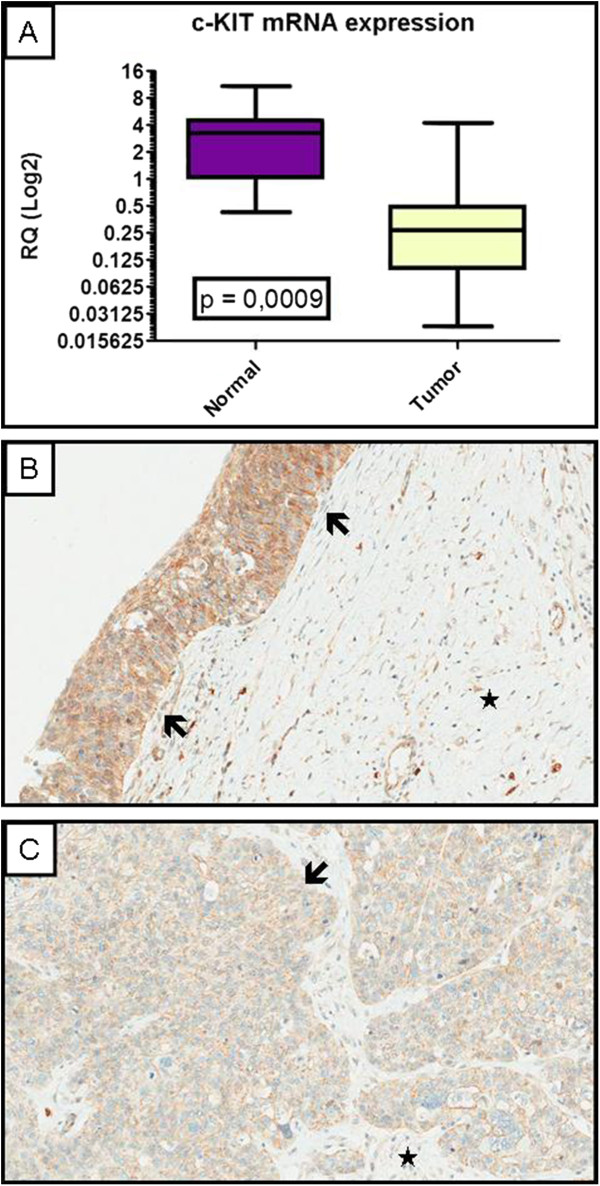
**c-KIT mRNA and protein expression in vulvar carcinomas.** (**A**) c-KIT mRNA expression evaluated by qRT-PCR using Relative Quantification (RQ) values (shown vertical axis). Normal samples transcript expression is shown significantly higher than tumor expression (p = 0.0009); c-KIT protein expression assessed by immunohistochemistry showing stronger immunostaining in normal area (**B**, arrows: normal epithelium, star: stroma) than in tumor area (**C**, arrow: invasive squamous cell carcinoma with irregular infiltrating borders, star: stroma).

*c-KIT* gene expression quantitated by qRT-PCR revealed higher levels of mRNA transcripts in normal samples (average relative quantification–RQ: 3.21) when compared to tumor samples (average relative quantification–RQ: 0.54), as shown on Figure4 (p = 0.0009). These observations could not be compared with immunohistochemistry and clinicopathological variables as the sample size was too small to perform statistical tests.

When evaluated by IHC in a smaller set of whole section tumors (10 cases), using IHC in whole section tumors, the protein product was also higher in normal areas as seen in Figure4B when compared to tumor areas from the same (Figure4B and C).

## Discussion

As vulvar carcinoma is a relatively rare cancer, very little is known about novel biomarkers with prognostic or predictive roles, although initial efforts are being made. Patients and health services must be made aware of the importance of gynecological evaluation and education policies should be strengthened to increase awareness not only about vulvar carcinoma, but also cervical cancer and HPV infection. In our study, we wished to determine the prognostic importance of c-KIT, a novel prognostic and predictive biomarker for other neoplasms, by evaluating its protein and mRNA expression in vulvar squamous cell carcinomas and correlating its positivity with clinicopathological features and HPV infection.

Our results showed a c-KIT protein positivity by IHC in 70.5% of cases, which was associated with a higher recurrence-free and global survival, an absence of associated lesions or lymph node metastasis and HPV infection, suggesting an important role of this receptor in vulvar carcinoma as a good prognosis marker. Furthermore, c-KIT mRNA quantitation revealed higher transcription levels in normal samples than tumor samples. Thus, this receptor may have potential as a good prognostic marker in vulvar carcinomas.

Proto-oncogene *c-KIT* encoded tyrosine kinase appears to have an important role in carcinogenesis of various tumors, including gastrointestinal stromal tumors (GIST), melanomas, seminomas, glioblastomas, breast cancer and acute myeloid leukemias (AML) [17,18]. The c-KIT/SCF system seems to be also involved in carcinogenesis of the female genital tract [23]. In most tumors, progressive decrease in c-KIT expression is reported as long as the tumor grows and invades [23,24], being positive in normal tissues and decreased in tumor cells. Such data corroborates with our study, since significantly higher expression of *c-KIT* mRNA was observed in normal tissue when compared to tumor microdissected samples.

In this study, high IHC expression of c-KIT receptor was similar to that described by Eroglu et al. (2008) [25] who found a strong diffuse high expression of protein in the cytoplasm and/or membrane of vulvar epithelioid sarcoma cells. The frequent finding of IHC cytoplasmic staining among our cases suggests an internalization of the receptor after its binding to SCF, which may indicate a physiological regulation of this receptor in the cells, the result of a rapid turnover of the protein when no longer located in the membrane [9,26].

Longer global and disease free survivals were observed in our patients with positive expression of c-KIT. Similar findings have also been shown by other authors in neuroblastomas [9], nasopharyngeal carcinomas [20] and multiple myeloma [21]. These authors reported that the expression of c-KIT and a better prognosis is an “unexpected” finding since, in several tumor types, c-KIT is associated with malignancy [9] as a result of constitutively turned on tyrosine kinase and, in the case of both expression of c-KIT and its ligand SCF, as a result of self-support from an autocrine tumor growth feedback loop [9,19]. Bataille et al (2008) [21] found a 93% survival in 4 years for patients with c-KIT positive multiple myeloma versus 64% for c-KIT negative. Similarly, our data show overall survival of 72% for c-KIT positive and 50% for c-KIT negative. Also, a correlation between c-KIT positivity and better recurrence-free survival was observed. Both vulvar skin bridge recurrences and primary tumor site recurrences have been shown in the literature as strong predictors for cancer-related death [27] and, therefore, this supports the utility of this marker as a powerful predictor for tumor recurrence.

The fact that c-KIT exerts divergent functions depending on its modulation by environmental factors, its interactions with several different intracellular effectors pathways and alternative splicing of its mRNA, may account for the differences in function found in different tumors [9,28,29]. Studies done in vitro and in vivo in melanoma cells have shown that exposure of c-KIT positive cells to SCF triggers apoptosis, which does not happen in c-KIT negative cells or in normal melanocytes. Since SCF is normally produced by keratinocytes and other dermal cells, loss of expression of c-KIT may allow malignant melanoma cells to escape c-KIT/SCF mediated apoptosis, contributing to growth and tumor metastasis [29,30].

The most important clinical prognostic factor in vulvar cancer is the lymph node status, which is a part of surgicopathological staging by FIGO [31]. In this sense, it is important to note that a grouping strategy regarding lymph node involvement was made to allow a biologically rational insight of the statistical analysis. Our results show that c-KIT positive cases were associated with absence or involvement of only one lymph node (p = 0.0053) when compared to c-KIT negative cases, which were associated with two or more lymph-node metastasis. This grouping strategy was used by Holschneider and Berek [32] and states that patients with no nodes or only one microscopically involved have a comparable prognosis. The association between positive staining for c-KIT and absence of lymph node metastases, absence of associated lesions, and higher survival rates indicates that this is a marker of good prognosis in vulvar cancer. Possibly, in tumors with positive staining for c-KIT, more conservative surgery with less mutilation should be evaluated in order to provide a better psychosocial quality of life of these women. These psychosocial issues are widely described in the literature as one of the most important concerns especially among younger patients, regarding complications of therapy [31]. Fear, distorted body image and depression, are the most frequent symptoms mentioned in the literature, in this regard [31]. Implementing translational research findings including molecular marker evaluation, such as c-KIT, could be helpful in developing a more personalized approach to the treatment of these patients. Thus, c-KIT positivity could be used as a prognostic factor for vulvar carcinoma.

A relationship between positive expression of c-KIT and the presence of HPV was demonstrated in this study, although there is no data in the literature suggesting this. We hypothesize that (1) the locus of *c-KIT* gene may be a fragile site yet unknown for integration of HPV; or (2) abnormalities in the regulation of this gene may be accelerating the process of carcinogenesis in cooperation with the viral infection. Besides, the positive correlation between c-KIT expression and HPV reinforces the good prognosis of these tumors, as tumors associated with HPV, which is considered an independent predictor of better survival [33].

Regarding treatment and further predictive values of c-KIT, the positive overexpression of this receptor was found to be associated with a better global and disease free survival in patients with vulvar cancer. For this reason, contrary to the situation found in other c-KIT positive tumors, the use of a c-KIT inhibitor such as Gleevec® may be inappropriate in vulvar cancer. However, a better understanding of the molecular regulatory mechanisms of c-KIT may, in the future, play a role in the development of targeted therapies in order to inhibit or modulate the signaling pathways triggered by this receptor.

Vulvar carcinoma is a rare disease, and thus our study was limited in size. We also encountered problems with lack of clinical information, which reduced numbers further. Scarcity of publications also leads us to a more speculative discussion and comparison with other neoplasms.

The association between positive staining for c-KIT and absence of lymph node metastases, absence of associated lesions, and higher survival rates indicates that this is a marker of good prognosis in vulvar cancer. Possibly, in tumors with positive staining for c-KIT, more conservative surgery with less mutilation should be evaluated in order to provide a better psychosocial quality of life of these women [31]. Fear, distorted body image and depression, are the most frequent symptoms mentioned in the literature regarding the psychosocial quality of life of these patients [31] and could be relieved if more personalized medicine through molecular markers evaluation was used. Thus, molecular marker evaluation (c-KIT positivity), could be used as a prognostic factor for vulvar carcinoma, implementing translational research findings into part of a more personalized approach to each individual patient and their therapy.

## Conclusions

The literature has already shown the important role of the tyrosine kinase encoded by *c-KIT* in carcinogenesis of various tumors. Our study suggests its important role in vulvar carcinoma as a marker for good prognosis. This implies that the most aggressive vulvar cancer cases may not benefit from c-KIT targeted therapies as they already have low c-KIT expression. The high expression of this receptor among our cases suggests that, a better understanding of the molecular mechanisms involved in patients with c-KIT positivity will be required to select a more appropriate type of target therapy. In summary, novel conservative surgical approaches may be possible after IHC evaluation of c-KIT in vulvar cancer, bringing results from bench to the bedside in order to provide a better psychosocial quality of life for the women.

## Abbreviations

AML: Acute myeloid leukemia; c-KIT: v-kit Hardy-Zuckerman 4 feline sarcoma viral oncogene homolog; FIGO: International federation of gynecology and obstetrics; FFPE: Formalin-fixed paraffin-embedded; GISTs: Gastrointestinal stromal tumor; HPV: Human papillomavirus; IHC: Immunohistochemistry; mRNA: Messenger RNA; qRT-PCR: Quantitative real-time polymerase chain reaction; RQ: Relative quantification; RR: Relative risk; SCC: Squamous cell carcinoma; SCF: Stem cell factor; SD: Standard deviation; TMA: Tissue microarray; VINs: Vulvar intraepithelial lesions; VSCC: Vulvar squamous cell carcinoma.

## Competing interests

The authors declare that they have no competing interests.

## Author’s contributions

*BMM* conceived the study design, participated in its design and in the acquisition of data, analysis and interpretation. *AMLR* participated in the acquisition of data, analysis and interpretation. *ISR* participated in the acquisition of data, analysis and interpretation. *GB* participated in the acquisition of data. *FMC* participated in the acquisition of data from medical records. *MMS* participated in the analysis and interpretation of the data. LTDC performed statistical analysis. *KCC* participated in the RNA extraction, qRT-PCR procedure and analysis. *FAS* has been involved in drafting the manuscript or revising it critically for important intellectual content. *RMR* conceived the study, participated in its design and coordination. All authors have given final approval of the version to be published.
